# The Plague

**Published:** 1900-08

**Authors:** 


### EDITORIAL NOTES AND COMMENTS.

---

The Business office of THE JOURNAL-RECORD is 305, 306 Fitten Building.

The Editorial office is 318-319-320 The Prudential.

Address all Business communications to Dr. M. B. Hutchins, Mgr.

Make remittances payable to THE ATLANTA JOURNAL-RECORD OF MEDICINE.

On matters pertaining to the Editorial and Original Communications address Dr. Bernard Wolff, Atlanta.

Reprints of original articles will be furnished at cost price. Requests for the same should always be made *on the manuscript*.

We will present, post-paid, on request, to each contributor of an original article, twenty (20) marked copies of THE JOURNAL-RECORD containing such article.

---

### THE PLAGUE.

THE history of outbreaks of grave communicable disease in the cities of this country has always shown that the boards of health and the sanitary authorities are the objects of lively criticism during such a time. Extreme caution is necessary before announcing the presence of the disease, in order that business interests may not be compromised by immature and precipitate conclusions. The situation at San Francisco reflects the difficulties in the way of those who have the responsibility of the public health entrusted to them during periods of great stress as that occasioned by the alleged presence of the plague, and it also illustrates by the rancorous factional contentions of the medical profession the incompleteness of diagnostic knowledge of the plague. We have before us copies of two influential medical journals published in San Francisco. One quotes from the report of Governor Henry T. Gage, that there is "no bubonic plague in San Francisco." The other deliberately asserts that the plague is now epidemic in the Chinese quarter, where there are 35,000 Chinamen more or less directly exposed. How on earth are these opposite statements to be reconciled? and what is the outside world to believe? The statement is made by one of these journals that competent medical men of unimpeachable integrity and ability examined every "suspected" case to which they could gain access. They were present at post-mortems, made extensive microscopic and bacteriologic researches, and came to the conclusion that no plague existed. The other journal states positively that there have been fourteen deaths from plague in San Francisco since March 16.

Whatever be the facts, one truth is apparent: The profession of San Francisco seems more concerned in airing its esoteric differences than in guarding the health of one of the most important ports of entry of the United States against the spread of a pestilence of whose presence there is at least reasonable ground for belief.

It would be a more gratifying sight to witness a strong, united effort to oust the infection than a polemic on paper and an abstract discussion of the relation of theory and practice. The plague is no theory, but a condition, a menace more or less imminent, which must be combated, not by words but by decisive action. It is not probable that it will gain a foothold in America, but the only thing that prevents it is vigilance and active interference. There is no natural reason of which we are aware that is antagonistic to a spread of the plague. It is the imperative duty of every seaport to use an unrelenting watch for signs of the disease on incoming ships. It is another way of saving the country, and any way is better than the puerile tit-for-tat contention running to-and-fro like a weaver's shuttle among the San Francisco doctors.

The occasion is preeminently one for organized work, not acrimonious criticism.

---

MANY people imagine that with the spread of science and religion disease and war will be abolished. No true lover of the race can look with complacency upon such a prospect. It is disease that makes the human race strong and healthy. Where are the most vigorous men and women to be found? The answer is, in those regions where consumption is most prevalent. In countries where consumption is unknown the race has physically deteriorated; but in countries like our own, where consumption is the most prevalent disease, we find the finest physical type of man. The reason is plain. Consumption kills off the feeble and leaves the strong to propagate the race. Abolish consumption, and the race will be propagated by the feeble to a greater extent than by the strong, since the former greatly outnumber the latter. In the course of a few generations the abolition of consumption in America would so diminish the stature and strength of Americans that we should rank physically very little above the Japanese. Disease is one of the conditions of the survival of the fittest. Abolish disease, and the weak and defective will have the same chances of life as have the strong. The unfittest will survive as certainly as the fittest, and the unfittest will be, as they naturally are, the majority.

The above is taken from a popular magazine of wide circulation and is quoted as an instance of inane twaddle that is dished out to the public as science. Such stuff is doubly objectionable, not alone because it is not true, but because it teaches a spirit of complaisant indifference to the advance of the fight that is being made against the spread of tuberculosis which has never been so vigorously waged as at the present.

What can the faithful workers for the good of humanity expect from the people when they are being urged on the contrary to an attitude of indifference little better than fatalism.

Disease, especially preventable disease, is not a necessity because it falls in an antithetic relation to health. Death alone is inevitable as the terminal stage of mundane existence, not as the closing picture of disease. To argue that the presence of consumption is desirable because it weeds out the unfit and thus strengthens the race is to argue what is not a fact. Tuberculosis is an infectious disease and bestows its infection impartially. The robust fall to it as well as the feeble. It has not such selective adaptability as claimed by the writer. His observations may be true of war, but they are absurd as applied to the rôle of disease in the development of a people.

